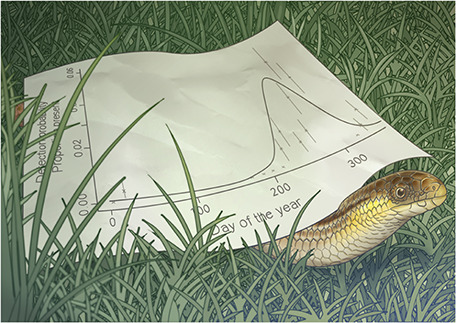# Where and when to look: conservation physiology can optimize monitoring surveys

**DOI:** 10.1093/conphys/coab005

**Published:** 2021-02-06

**Authors:** Sean Tomlinson

**Affiliations:** School of Biological Sciences, Benham Laboratories, The University of Adelaide, North Terrace, Adelaide, SA 5005, Australia

Conservation of rare or threatened species can be complicated under the best circumstances—sometimes just simply *finding* the species to assess their populations is difficult enough, let alone monitoring their responses to management or recovery plans. Moreover, survey efforts are often hugely expensive and not always efficient at recording the target species. Kate Saleeba and her colleagues saw this as a problem for the cryptic, striped legless lizard (*Delma impar*), a species that is currently threatened with extinction. Saleeba’s team aimed to address this problem by understanding more about when and where these lizards are active using biophysical ecology, so that conservation managers could more successfully find and protect them.

There is a growing recognition that the rising biodiversity crisis will demand hard decisions. Questions have been rising and will continue to do so. Which species need to be actively managed? Which species need to be conserved? Where should efforts be exerted to achieve the best ‘bang for buck’? Yet, these decisions cannot be made without baseline data on the species, which may be challenging for those that are cryptic or those that occur patchily in space and time. With the growing number of cryptic species represented on threatened species lists, a blitzkrieg of general assessment surveys is no longer an option; more targeted survey efforts are required.

In this space, Saleeba and her colleagues propose an elegantly simple solution: establish a quantitative, physiologically informed model of when and where your species is most likely to be active and observable, and only survey for your species in those times and spaces. Sounds simple, right? Saleeba and her colleagues collected physiological data describing the thermal preferences and tolerance limits of their target species using a passive thermal gradient and some low-temperature incubators, which are both cost-effective and minimally invasive. Yet, this approach can help develop remarkably powerful and insightful models to guide survey efforts and potentially other conservation activities. The model developed by Saleeba and colleagues based on these critical temperatures proved very accurate in predicting the absence of striped legless lizards. It did, however, also tend to incorrectly over-predict the presence of the lizards. In the context of optimizing a survey program, however, it is much more valuable to be spot-on regarding lizard absences because it prevents the waste of survey effort in times and places where lizards are not active. Alternatively, it is much better to over-survey in times and places where the lizards *might* be active to maximize the chance of finding them.

Engagement within the conservation movement, including allocating resources and spending, on physiology and biogeography studies continues to be a challenge. Conservation agencies and charities often prefer to spend money on practical conservation, such as active survey work, rather than engaging in physiological research that feels pretty vague and obscure. Quite often, this can lead to inefficiency and poor conservation outcomes. Saleeba and colleagues contribute sound experimental physiology with practical objectives to illustrate how an apparently obscure understanding of lizard physiology and thermal tolerance can be used to optimize survey effort for this and other cryptic organisms. It is precisely this kind of evidence-driven approach that is essential in meeting the rising challenge of the biodiversity crisis of the Anthropocene.

Section Editor: Jodie L. Rummer

Illustration by Erin Walsh; Email: ewalsh.sci@gmail.com